# Artificial Intelligence for STN-DBS Surgical Planning in Parkinson’s Disease: A Multicenter Study Comparing Conventional Targeting Versus Supervised Statistical Machine Learning

**DOI:** 10.3390/brainsci16050457

**Published:** 2026-04-24

**Authors:** Feifei Wu, Raffaella Buonanno, Valentina Baro, Vincenzo Levi, Giulia Melinda Furlanis, Mariasole Gagliano, Andrea Guerra, Alberto D’Amico, Carlo Giorgio Giussani, Roberto Eleopra, Luca Denaro, Angelo Antonini, Andrea Landi

**Affiliations:** 1 Academic Neurosurgery, Department of Neuroscience, University of Padua, 35128 Padua, Italy; feifei.wu@studenti.unipd.it (F.W.); valentina.baro@aopd.veneto.it (V.B.); furlanis.giuliamelinda@gmail.com (G.M.F.); mariasole.gagliano@studenti.unipd.it (M.G.); alberto.damico@unipd.it (A.D.); luca.denaro@unipd.it (L.D.); 2 Unit of Neurosurgery, School of Medicine and Surgery, Department of Medicine and Surgery, University of Milano Bicocca, 20126 Milan, Italy; raffaella.buonanno@asst-fbf-sacco.it (R.B.); carlo.giussani@unimib.it (C.G.G.); 3 Functional Neurosurgery Unit, Neurosurgery Department, Fondazione IRCCS Istituto Neurologico Carlo Besta, 20133 Milan, Italy; vincenzo.levi@istituto-besta.it (V.L.); roberto.eleopra@istituto-besta.it (R.E.); 4 Neurodegenerative Diseases Unit, Department of Neuroscience, University of Padua, 35128 Padua, Italy; andrea.guerra@unipd.it (A.G.); angelo.antonini@unipd.it (A.A.); 5 Padua Neuroscience Center, 35128 Padua, Italy; 6 Functional Neurosurgery Unit, Department of Neuroscience, University of Padua, 35128 Padua, Italy

**Keywords:** AI, DBS, Parkinson’s disease, functional neurosurgery

## Abstract

**Highlights:**

**What are the main findings?**

- In this multicenter series of bilateral STN-DBS implantation, AI-based targeting showed agreement with conventional stereotactic and MER-guided along the lateral (X) axis.
- Significant differences emerged along the antero-posterior (Y) and cranio-caudal (Z) axes, reflecting methodological and intraoperative factors rather than systematic AI inaccuracy.

**What are the implications of the main findings?**

- AI-based planning may contribute to standardizing STN targeting and reducing operator-dependent variability in DBS surgery.
- Predictive machine learning tools may have the potential to assist in asleep-DBS procedures and help reduce the need for intraoperative microelectrode recordings.

**Abstract:**

**Objective**: Deep Brain Stimulation (DBS) has been consolidated as a valid therapeutic option for advanced Parkinson’s disease (PD). The identification of specific targets can be achieved through different methods, including conventional direct and indirect methods. The aim of our multicentric study is to provide a comparison between these traditional methods and artificial intelligence (AI) in the ascertainment of the ideal targets. **Materials and Methods**: A total of eight patients, who received bilateral STN (subthalamic nucleus) DBS implantation between 2022 and 2023 were analyzed. Target coordinates were calculated based on the Schaltenbrand and Wahren atlases and the AI using the RebrAIn system during the planning phase; intraoperatively, the targets were either confirmed or adjusted according to microelectrode recordings (MERs). The differences in the three Cartesian axes of stereotactic coordinates (X, Y, and Z) according to these methods were evaluated and compared through non-parametric ANOVA Friedman test. **Results**: The results revealed significant agreement in the lateral–lateral coordinates (X, X′, X″), indicating stability in target determination along this axis across the methods. However, more substantial discrepancies were observed in the antero-posterior and cranio-caudal coordinates, suggesting lower consistency between the examined methodologies. **Conclusions**: Our preliminary study results suggest that, despite the challenges posed by interindividual anatomical variability and the limitations of imaging techniques, artificial intelligence has shown comparable values on the lateral–lateral X coordinates. The accuracy of predictive targeting using machine learning models needs to be validated by further studies, but the preliminary results appear to indicate a potential promising role for artificial intelligence in integrating the preoperative workflow.

## 1. Introduction

Deep Brain Stimulation (DBS) has become an established therapeutic option for the treatment of carefully selected Parkinson’s disease (PD) patients.

The procedure involves the implantation of electrodes in the basal ganglia using a stereotactic technique. It can be tailored to the patient’s symptoms by targeting different areas along the cortico-basal circuit, which mediates various symptoms of the disease [1,2,3]. DBS in the thalamus (VIM) is particularly effective for tremors, while stimulation in the internal globus pallidus (GPi) is more beneficial for reducing rigidity and dyskinesias. In contrast, targeting the subthalamic nucleus (STN) can address tremors, akinesia, rigidity, and dyskinesias. Additionally, STN-DBS allows for a reduction in medication doses, even in patients with advanced stages of the disease, making it the preferred target for this condition [4,5].

The use of ventriculography has enabled the development of stereotactic atlases, which form the basis of the indirect method (i.e., the identification of nuclei without direct imaging of them) [6]. This approach establishes coordinates that statistically reflect the position of the targets relative to the AC-PC line. Intraoperative X-ray and microelectrodes recordings (MERs) are used to verify the correct placement of the electrodes.

With the introduction of CT and later MRI, including volumetric sequences, an alternative technique known as the direct method emerged. This approach allows direct identification of the targeted nuclei. During the intraoperative phase, this pre-surgical preparation is complemented by microelectrode recordings (MERs) of the selected target and/or a clinical assessment of the patient. These additional measures help to reduce risks and enhance the accuracy of electrode placement [7,8]. To minimize potential complications and patient discomfort associated with the procedure, and with the advancements in neuroimaging enabling more precise visualization of the nuclei, an increasing number of centers have transitioned to performing surgery under general anesthesia (asleep surgery). As a result, the use of MERs and intraoperative clinical evaluation has gradually declined [9].

However, discrepancies have been noted between the STN identified on various MRI sequences and perioperative electrophysiological findings. This inaccuracy is primarily attributed to the absence of standardized techniques, making the results highly dependent on the operator.

Still, there is currently no compelling scientific evidence to prove that one methodology is more effective than another [10,11,12].

Given current trends, one potential approach to standardizing the procedure could be the use of machine learning systems to reduce variations among surgeons during surgical planning. Artificial intelligence (AI) has a growing relevance in neurosurgical procedures [13]. Specifically, the PARKEO2 study protocol [14] employs a supervised statistical learning system (RebrAIn © [15]). For this purpose, patients with PD who showed significant improvement following STN-DBS were first identified. Subsequently, the active contacts of their electrodes and eighteen anatomical landmarks surrounding these contacts were determined on a 1.5T MRI. Diffusion tensor imaging tractography was also included to avoid damage to the fiber tracts. These landmarks were correlated with the coordinates of the active contacts, using a statistical machine learning system, and used as a reference to predict the optimal STN target based on clinical outcomes, with the aim of guiding DBS targeting in new PD patients selected for the procedure.

The objective of our study is to compare our case series of DBS electrode implantation in the STN, using conventional target localization based on atlas coordinates plus direct nuclei visualization and MERs, with the target provided by artificial intelligence, to demonstrate the correlation between the neurophysiological sweet point, the optimal electrical target, and the AI-generated target.

In 2022, the center of Pediatric and Functional Neurosurgery Unit in Padua had the opportunity to use the RebrAIn © OptimDBS software for a limited trial as a preliminary testing of this method.

This preliminary study aims to calculate the difference in target identification in STN-DBS for patients with Parkinson’s disease, comparing three methods:

1. The conventional method used in our center, which applies the Schaltenbrand and Wahren [6] coordinates adjusted with 3D MRI imaging (aided by Brainlab^®^ software).
2. The actual postoperative targets identified through microelectrode recordings (MERs).
3. The targets identified by RebrAIn © software

If these three methods prove to be comparable, artificial intelligence could standardize a procedure that is currently operator-dependent. This could potentially eliminate the intraoperative MER phase, making the procedure not only faster and safer but also more feasible in centers with less experience in intraoperative micro-recordings.

The research was conducted in accordance with the Local Ethical Committee rules, following the criteria of the Helsinki conference.

## 2. Materials and Methods

This study, following the same premises and procedures, was conducted in collaboration with the Functional Neurosurgery Unit of the Carlo Besta Neurological Institute (Milan, Italy).

The study obtained the license from the Ethic Committee of our institution (Study: COORDINATES-DBS. Code CET-ACEV: 6413n/AO/25, on 2 October 2025).

### 2.1. Data Collection

In 2022, our center enrolled five patients with Parkinson’s disease (PD) in our study, all of whom underwent STN-DBS surgery. In 2023, the Carlo Besta Neurological Institute included three patients with PD and one with PKAN syndrome in its study, all of whom underwent STN-DBS surgery. In the current study only patients affected by PD were considered. The availability of the RebrAIn © software was limited to a trial period for a preliminary testing.

Prior to each procedure, patients underwent an initial neurological and neurosurgical evaluation. Following a follow-up period, a neurosurgical eligibility assessment was performed to determine the surgical indications for Deep Brain Stimulation. If the patient was considered a suitable candidate, the study’s inclusion and exclusion criteria were then assessed.

### 2.2. Inclusion Criteria

- Patients diagnosed with Parkinson’s disease who are eligible for STN-DBS surgery (CAPSIT criteria [16]);
- Age between 18 and 70 years;
- Patients capable of providing informed consent.

### 2.3. Exclusion Criteria

- Patients who have experienced major complications from STN-DBS surgery (such as ischemia or hemorrhage);
- Patients with severe psychiatric disorders;
- Patients with dementia;
- Patients with preoperative clinical and radiological signs of ongoing or severe cerebrovascular disease, neoplastic pathology, or cerebrospinal fluid dynamic disorders;
- Patients with a cardiac pacemaker.

### 2.4. Preoperative Study

**Figure 1:**
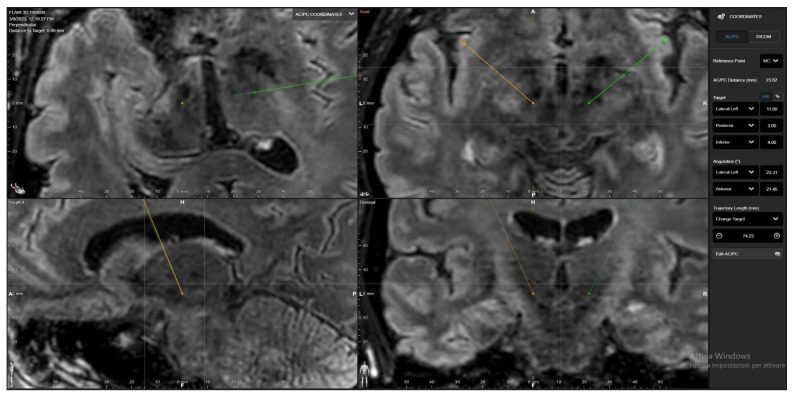
Conventional targeting using Schaltenbrand and Wahren [6] coordinates adjusted with 3T MRI imaging.

The study population underwent preoperative neuroradiological evaluation. At our center, a 3T brain MRI, including T1w and FLAIR sequences with and without contrast, was performed, incorporating complete preoperative volumetric sequences. The AC-PC line and the intercommissural midpoint were visually identified using Brainlab^®^ Stereotaxy software. Target coordinates were calculated based on the Schaltenbrand and Wahren [6] atlases (from the intercommissural midpoint: 11 mm laterally, 3 mm posteriorly, and 4 mm inferiorly), and the two targets were subsequently identified. Figure 1.

**Figure 2:**
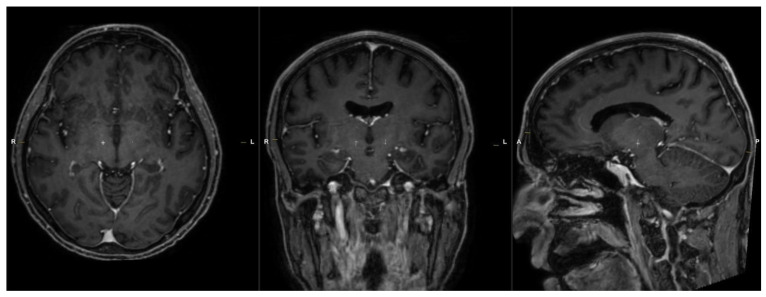
RebrAIn coordinates calculated on the same preoperative 3T MRI. The targets are indicated by the white cross.

Intraoperatively, MRI fusion with CT after application of a stereotaxic frame was conducted, the targets were either confirmed or adjusted according to the results of microelectrode recordings (MERs). On average three MERs passes per side were performed per side. The results of the MERs were interpreted by expert neurosurgeons and neurologists. At the same time, the DICOM files of the preoperative T1w-volumetric MRI sequence were transmitted to the RebrAIn OptimDBS © software for target calculation using artificial intelligence, which returned the results in DICOM format. Figure 2.

### 2.5. Postoperative Study

**Figure 3:**
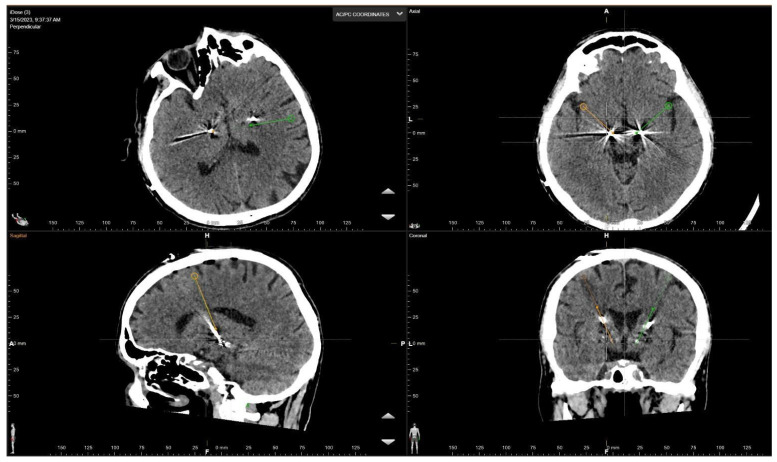
Postoperative CT scan showing the final position of the electrodes obtained after microelectrode recordings.

Postoperative neuroradiological assessment (brain CT) was conducted to confirm the accurate placement of the stimulating electrodes and to detect any acute complications. The coordinates of the actual target were subsequently recalculated and compared with those derived from preoperative planning as well as those identified by artificial intelligence (using Brainlab^®^ Stereotaxy software). Figure 3.

The differences in the three Cartesian axes of the preoperative stereotactic coordinates (X, Y, and Z), those modified based on intraoperative MERs (X′, Y′, Z′), and those determined by artificial intelligence (X″, Y″, Z″), respectively called “stereotactic”, “electrophysiological”, and “AI” coordinates, were then calculated.

## 3. Results

A total of eight patients who underwent bilateral STN-DBS implantation between 2022 and 2023 were analyzed. The left and right sides of each patient were considered independent variables. Therefore, the results were analyzed on an actual statistical population of sixteen sides (Table 1). The numbers in the table refer to the stereotactic coordinates calculated based on the AC-PC line, in millimeters. The “X” values define the laterality of the target concerning the AC-PC line from the MCP. The “Y” values represent the antero-posterior distance (positive numbers indicating a position anterior to the AC-PC midpoint and negative numbers indicating a position posterior to it). The “Z” values indicate the cranio-caudal distance (with positive numbers representing a position above the plane containing the AC-PC line with respect to the external stereotactic frame reference and negative numbers representing a position below to it).

We compared the three independent measurements taken from the same patients (with double-blind repeated measurements obtained by different observers) by performing an analysis of variance on the medians of the three groups, accounting for the dependence between the measurements. Considering the small population and the non-normal distribution of the data, it was not possible to use a parametric test and therefore the Friedman test was used, which is the non-parametric version of the ANOVA.

The hypotheses formulated were as follows:

- Null hypothesis (H0): the medians of the three measurements are equal;
- Alternative hypothesis (H1): at least one of the three measurements is different from the others.

The Friedman test for the comparison of measurements X, X′, X″ returned 4.72 as result (*p*-value 0.094, α = 0.05) and so it is not possible to reject the null hypothesis. This indicates that there is insufficient evidence to conclude that a significant statistical difference exists between the three measurements on the X coordinate, suggesting that the stereotactic, electrophysiological, and AI targets are statistically comparable.

The Friedman test for the Y, Y′, and Y″ measurements yielded a result of 14.70 (*p*-value = 0.00064, α = 0.05). Since the *p*-value is significantly lower than the typical significance level of 0.05, we can reject the null hypothesis. This indicates that there is a significant difference between the three measurements.

The Wilcoxon post hoc test was then performed to compare the pairs of measurements which yielded the following results:

- Comparison between Y and Y′: 27.0 (*p*-value 0.0608, α = 0.05), not significant;
- Comparison between Y and Y″: 22.0 (*p*-value 0.0155, α = 0.05), significant;
- Comparison between Y′ and Y″: 16.0 (*p*-value 0.0052, α = 0.05), significant.

The Holm–Bonferroni Correction test was carried out to take into account for Family-Wise Error Rate (FWER) yielding the following adjusted *p*-values respectively: 0.0608, 0.0156, 0.0310, α = 0.05.

Thus, a significant statistical difference exists between Y and Y″, as well as between Y′ and Y″, while no difference is observed between Y and Y′. Therefore, we can conclude that the AI target (Y″) is significantly different from the stereotactic and electrophysiological targets on this coordinate.

Finally, the Friedman test for the Z, Z′ and Z″ measurements returned 20.86 as results (*p*-value 0.00003, α = 0.05). This suggests that there are statistically significant differences between at least one of the three measurements.

The post hoc analysis with the Wilcoxon test produced the following results:

- Comparison between Z and Z′: 4.0 (*p*-value 0.00147, α = 0.05), significant;
- Comparison between Z and Z″: 10.5 (*p*-value 0.00131, α = 0.05), significant;
- Comparison between Z′ and Z″: 4.0 (*p*-value: 0.00021, α = 0.05), significant.

The Holm–Bonferroni Correction test was applied giving the following adjusted *p*-values: 0.00063, 0.00262, 0.00262, α = 0.05.

This implies that, for this coordinate, all three targets are statistically and significantly different from each other.

### Clinical Outcome

All patients tolerated well the surgical procedure; no complications were registered for the eight patients. The clinical follow-up was performed as usual 30 days after surgery, then DBS was activated after careful adjustment of the stimulation parameters. No patient experienced collateral effects or worsening of the pre-surgical clinical status at the stimulus thresholds for positive effects in each tested contact after monopolar review. According to standard stimulus parameters (monopolar stim, 60 microsec duration, 130 Hz), the thresholds for positive effects were comparable to those obtained in patients operated in the conventional way. After three months of FU, all the patients were evaluated according to the CAPSIT protocol [16], using MDS-UPDRS [17]. Global UPDRS-III section (motor section) decreased by 33% on average, obtaining the best performances in tremor control, and stiffness reduction. Moreover, dyskinesias promptly decreased and drug intake was reduced by 20% in five patients. Further FU at six months confirmed the positive trends both in clinical improvement and in drug withdrawal. Clinical data were comparable with the results obtained in our series of patients who underwent STN-DBS in conventional way [18,19].

## 4. Discussion

Determining the optimal target for Deep Brain Stimulation (DBS) has been the subject of debate since the introduction of this surgical technique, due to the complexity of brain anatomy and the need for precise electrode placement to maximize therapeutic efficacy and reduce side effects. Accurate positioning is essential, as small errors can cause side effects such as muscle twitching, speech disturbances, and gaze deviation [20,21]. Traditionally, DBS targeting has been based on different methodologies, including indirect stereotactic targeting, which uses standardized anatomical references with respect to the AC-PC line, electrophysiological targeting, which uses intraoperative microelectrode recording (MER) to identify the neurophysiological characteristics of the target structures, and more recently, AI-supported clinical targeting, which relies on advanced predictive models to define the optimal electrode position.

The use of a standardized method for targeting STN-DBS has also been considered by other studies.

Guo et al. [22] proposed a system that uses functional 3D probabilistic maps built on the basis of actual stimulation data and intraoperative electrophysiological activity. These maps were integrated into software to automatically predict the optimal target and trajectory before surgery. The system uses advanced image registration and deformation methods to adapt the standardized data to the patient-specific brain. The method was assessed on ten DBS interventions in the subthalamic nucleus (STN), comparing the results with those obtained by an experienced neurosurgeon. The mean distance between the automatic target and the one established by the surgeon was 1.82 mm (±0.77 mm), indicating a high accuracy. The difference between the trajectories was 3.1° (antero-posterior) and 2.3° (lateral-medial). The functional map of the STN showed a spatial correlation of 80% with the actual target.

Kim et al. [23] proposed a machine learning-based (7T-ML) automatic segmentation model, trained on 7T images but applicable to 3T clinical images. The study compared the segmentation of the STN obtained using the 7T-ML model and that derived from standard atlases with the ground truth, defined as expert-validated manual segmentations, calculated on clinical data from 80 patients (160 STNs). The 7 T-ML showed significant superiority over standard atlases, with a mean center of mass distance (CMD) of 1.25 ± 0.60 mm, compared to higher values for other methods (2.37 mm to 4.25 mm, *p* < 0.0001, ANOVA). A total of 89.4% of STNs segmented with the 7T-ML had a CMD less than 2 mm, compared to much lower percentages for standard atlases. The mean distance between the active electrodes and the STN center of mass was significantly smaller in the 7T-ML (2.4 mm), much closer to the ground truth than in the atlases (3.2–5.6 mm). The 7T-ML model has been shown to be more accurate, consistent, and clinically useful than standard atlases for STN segmentation. Its high accuracy may improve targeting for DBS by reducing the risk of electrode placement errors.

Shamir et al. [24] continued this line of research by validating the clinical accuracy of the 7T-ML method by comparing it with intraoperative data obtained via microelectrodes (MERs), considered the gold standard for localizing the STN. All electrodes that intersected the STN by MERs also intersected the STN by the 7T-ML method. The mean STN length was 6.2 ± 0.7 mm by MERs and 5.8 ± 0.9 mm by the 7T-ML method. 93% of the electrode contacts were correctly classified by the 7T-ML method compared to the MER data.

Our multicenter study aimed to compare the superimposability of these three approaches by analyzing the stereotactic coordinates resulting from each targeting method. The results obtained show a significant agreement between the three methods in the lateral–lateral coordinates (X, X′, X″), suggesting that the target determination along this axis is stable among the different approaches. However, for the antero-posterior and cranio-caudal coordinates, more marked discrepancies emerged, indicating lower consistency between the methodologies examined. A possible explanation for this divergence lies in the distortion of magnetic resonance images (MRI), which tends to manifest more significantly along the antero-posterior and vertical axes, compromising the precision of targeting based exclusively on anatomical data [25,26].

Furthermore, in our clinical practice, among the best positions selected by the MER, the electrode is intentionally positioned with a slight posterior and caudal deviation from the theoretical target to maximize functional interaction with the somatosensory part of the subthalamic nucleus. This strategy is implemented by the presence of multiple contacts along the electrode, which allow greater flexibility in selecting the optimal stimulation field. Furthermore, the impact of intraoperative phenomena such as progressive CSF loss or the occurrence of peri-electrode edema [27,28,29] should not be underestimated, as they can determine a slight dislocation of the brain structures, modifying the actual position of the target compared to that planned preoperatively.

## 5. Conclusions

The results of this preliminary study suggest that, despite the complexities related to interindividual anatomical variability and the limitations of imaging techniques, artificial intelligence could be a useful tool for determining the stimulation target of STN-DBS. The accuracy of predictive targeting based on machine learning models appears to be comparable on the X axes to that obtained with traditional methodologies. With continued advancements in machine learning models, this comparability may further improve. Furthermore, according to preliminary studies [30], the surgical approach using artificial intelligence (in the absence of MER and with the patient asleep) seems to have a completely satisfactory clinical result that is comparable to other methods.

The integration of artificial intelligence in the preoperative workflow could represent a significant step towards the standardization of the STN-DBS procedure, optimizing the targeting process and making it more accessible even to centers with less experience in the method. A further advantage would be the possible elimination of the intraoperative microelectrode registration phase, with a consequent reduction in surgical time, risk of intraoperative complications and overall costs of the procedure, without compromising the precision of electrode positioning. However, it is necessary to underline that the current results are based on a limited number of patients and require further confirmation through multicenter studies on larger cohorts. The main limitation of the study is the small sample size which reduces the robustness of the statistical inference.

Future developments will also need to evaluate the long-term clinical impact of AI-based targeting, analyzing therapeutic responses in terms of efficacy and duration of stimulation, as well as the occurrence of any adverse effects related to electrode positioning. If these results are confirmed, the use of predictive algorithms could lead to greater uniformity of surgical outcomes and a better quality of life for patients with Parkinson’s disease, offering a less invasive, more efficient and potentially customized approach based on the specific characteristics of each individual.

## Figures and Tables

**Table 1:** Coordinates calculated according to the different methods.

Patients	X	X′	X″	Y	Y′	Y″	Z	Z′	Z″
P1 L	10.57	9.24	10.39	−2.87	−3.84	−1.91	−5.08	−7.25	−3.99
P2 L	11.32	12.49	12.12	−2.99	−3.47	−1.66	−3.3	−5.78	−0.67
P3 L	11.6	12.93	12.59	−3.32	−4.92	−1.38	−3.64	−5.85	−3.74
P4 L	9.84	10.61	12.06	−3.22	−3.99	−0.65	−3.81	−5.42	−2.02
P5 L	10.91	10.91	11.88	−2.09	−2.31	−5.44	−4.16	−7.66	−3.59
P6 L	11.51	11.61	12.21	−2.03	−2.39	−0.79	−3.99	−4.09	−2.09
P7 L	12.1	12	12	−2.9	−2.9	−2.5	−3.99	−4	−2.99
P8 L	10.99	11.33	13.77	−1.85	−2.33	−1.99	−3	−4.34	−3.34
P1 R	11.38	10.78	12.17	−1.91	−3.36	−2.69	−3.9	−7.42	−3.22
P2 R	10.68	10.75	11.23	−2.76	−1.7	−2.32	−3.67	−6.89	−1.78
P3 R	12.31	14.65	11.77	−5.38	−2.93	−2.31	−3.71	−6.09	−1.97
P4 R	11.15	12.41	12.72	−3.19	−4.53	−2.37	−3.2	−4.83	−2.56
P5 R	10.97	12.08	11.2	−4.13	−4.58	−2.72	−3.55	−6.65	−3.21
P6 R	12.37	12.3	11.91	−3.48	−3.38	0	−4.04	−4.09	−2.09
P7 R	12	11.95	12	−3.1	−3.4	−2.5	−4	−4	−2.99
P8 R	10.01	10.3	12.14	−1.84	−2.42	−1.64	−3.8	−3.34	−4.6

Footnotes: L: left side; R: right side; X, Y, Z: preoperative stereotactic coordinates; X′, Y′, Z′ actual postoperative coordinates modified according to intraoperative MERs; X″, Y″, Z″: clinical coordinates calculated according to artificial intelligence. The numeric value of the coordinates is expressed in millimeters.

## Data Availability

The data presented in this study are available from the corresponding author upon reasonable request. The data are not publicly available due to privacy and institutional restrictions.

## Abbreviations

The following abbreviations are used in this manuscript:

DBS	Deep Brain Stimulation
PD	Parkinson’s disease
AI	Artificial intelligence
STN	Subthalamic nucleus
MERs	Microelectrode recording
VIM	Thalamic ventral intermediate nucleus
GPi	Internal globus pallidus
AC-PC	Anterior commissure-posterior commissure
CAPSIT	Core assessment program for surgical interventional therapies in Parkinson’s disease
CMID	Center of mass distance
7T	7-Tesla MRI
CSF	Cerebrospinal fluid
MDS-UPDRS	Movement Disorders Society-Unified Parkinson’s Disease Rating Scale
